# Investigation of the thermal and physicochemical behavior of two types of gutta-percha cones for back-filling the root canal

**DOI:** 10.4317/jced.60095

**Published:** 2023-05-01

**Authors:** Jeannette Aguilar-Hernández, Fernando Torres-Méndez, María-Verónica Méndez-González, Claudia-Edith Davila-Perez, Eleazar-Samuel Kolosovas-Machuca, Mariana Gutierrez-Sánchez

**Affiliations:** 1 Master’s student in endodontics, Endodontics Postgraduate Program, Faculty of Stomatology, Autonomous University of San Luis Potosí, San Luis Potosi, Mexico; 2 Researcher-Professor, Endodontics Postgraduate Program, Faculty of Stomatology, Autonomous University of San Luis Potosí, San Luis Potosi, Mexico; 3 Researcher-Professor, Coordinación para la Innovación y Aplicación de la Ciencia y la Tecnología, Autonomous University of San Luis Potosí, San Luis Potosi, Mexico

## Abstract

**Background:**

Gutta-percha (Gp) is an inert thermoplastic polymer used as a filling to replace the dental pulp space, which has been reformulated to improve its three-dimensional sealing properties. Therefore, this study aimed to analyze the physical, chemical and thermal properties of two types of gutta-percha filling. As well as measuring the temperature distribution along the cone at the time of cutting through an in-situ test.

**Material and Methods:**

Two commercially available brands of gutta-percha point were investigated: Conform Fit TM Gutta-Percha for ProTaper Gold® (PTG) (Dentsply Sirona), and Hygenic Gutta-Percha (Coltene whaledent). Differential scanning calorimetry (DSC) and thermogravimetric analysis (TGA) were selected for the thermal characterization of materials, and Fourier Transform Infrared Spectroscopy (FT-IR) for the chemical analysis of Gp cones. Regarding temperature distribution, it was evaluated using a thermographic camera (FLIR ONE® PRO by MicroUSB P/N 435-0011-01) at 0 to 20 s after the cutting process (n=11/group).

**Results:**

Both materials have three fusion endotherms associated with the three crystalline phases of Gp, with similar temperatures but enthalpies that differ by 60%, the fusion enthalpy being higher for Conform Fit. In the chemical characterization, elements such as Zn, C, O, Ba, S and Si were found in both materials but in different proportions. Regarding the content of fillers, the Conform Fit presented around 30% of Gp polymer and 25% for the Hygenic. The morphological characterization shows a microtexturized coating in the form of bars on a micrometric scale for the Conform Fit, which could favor a better three-dimensional seal. In addition to that, in heat transfer studies they showed greater temperature control.

**Conclusions:**

The characterization of the materials allowed us to see the variation in terms of their composition and configuration to the Gp cones of two commercial brands. These variations directly modify the thermal behavior of the material.

** Key words:**Gutta-percha, Conform Fit, Infrared thermography, Differential Scanning Calorimetry, Infrared Spectroscopy.

## Introduction

In dentistry, the treatment of the root canal system consists of removing the pulp affected with irreversible damage and sealing the root canal with filling material that allows a three-dimensional seal that lasts and does not allow the subsequent entry of microorganisms. In this sense, gutta-percha (Gp) has been used for more than 100 years due to its good clinical performance. Gp can be considered the gold standard and currently the only universally accepted filling material (1-3). However, the incorporation of additives has been implemented to improve its properties since Bowman’s Gp introduction in endodontics in 1867. Gp is a word derived from the Malay language where gutah means rubber and pertjah, and is a purified coagulate made from the latex of a tropical sapote tree (1,4). Concerning the chemical structure, Gp is a poly (trans-1,4 isoprene) thermoplastic polymer that can exist in three crystalline forms known as α (alpha), β (beta), and γ (gamma), associated with the stereochemistry of its spatial configuration between the methyl groups (-CH3) that are attached to the carbon atoms with double bonds, leading to different spatial conFigurations that give it specific characteristics. The composition of dental Gp is approximately 18 to 22% of Gp polymer, and 37 to 75% filled with zinc oxide (ZnO) and barium sulfate (BaSO4); with waxes, colorants, and antioxidants that differ according to each manufacturer (5,6). Gp also has some advantages in the area of dentistry as it is inert, dimensionally sTable, and antibacterial, in addition to not staining dentin, being radiopaque, and dissolving in the presence of organic solvents such as xylol, xylene, and eucalyptol (7), allowing removal from the canal when necessary. However, Gp points lack rigidity, do not adhere to dentin, and some elasticity, resulting in gaps between the filling materials and the canal wall. Hence, sealant cement is necessary to ensure the apical sealing process’s success. In addition, some dentists, during treatment, heat the Gp tips with an obturator from the upper end to increase the fluidity of the material and provide a better seal within the canal system with complex anatomies. Previous thermal analyses reported Gp phase transformations to occur between 42 and 49°C for the beta phase and between 53 and 59°C for the alpha phase (6,8). In such a way, the material has to be heated to a temperature much higher than 60°C in order to cut the material and transfer the heat along the cone, which is affected by the material’s low thermal conductivity. Nevertheless, the limitations in the shutter temperature would be defined by the decomposition temperature of the material and the distance in which the heat is transferred along with the material, which could generate damage to the periodontal ligament (9,10). However, it should be noted that this mechanism is still unknown, so this study aimed to perform a thermal and physicochemical characterization and evaluate through in situ analysis how these heat transfer phenomena occur, and compare the Gp Conform Fit cones (Dentsply Sirona, that offers a new microtextured formula, and operates at a lower working temperature) with conventional Gp cones (Hygienic in this case).

## Material and Methods

-Materials

The sample used in this work is Gp Protaper Universal-Conform Fit (Dentsply Sirona Tulsa Dental), with a lot number. 8072809C, measured ISO 60xF2, Expiration 2022-09; and the Gp Hygenic (Coltene whaladent) batch. H09223, medium ISO 40, expiration 2022-11-30. All samples were analyzed before the expiration dates established by the manufacturers (n=19/group; 11 for the heat transfer test in situ, and the rest to analyze the physical, chemical and thermal properties).

-Chemical Characterization

The chemical characterization of the Gp was carried out by Fourier Transform Infrared Spectroscopy (FTIR) (Thermo Scientific, Nicolet iS10; Waltham, MA, US) using the attenuated reflection technique (ATR) in the range of 4000 to 500cm-1 with 64 scans at a resolution of 4cm-1. The samples were cut transversely for analysis.

-Thermal Characterization

Differential scanning calorimetry (DSC) and thermogravimetric analysis (TGA) were selected for the thermal characterization of the materials. The first with the aim of determining the characteristic temperatures of materials such as melting temperature (Tm), crystallization temperature (Tc), and glass transition temperature (Tg). TGA to determine the decomposition temperature and the content of charge. For DSC analysis, samples were taken from the last 3mm of the cone with an approximate weight of 9mg and analyzed in a Q200 DSC-TA-Instruments (Lukens Dr, New Castle, USA) instruments unit with a Nitrogen Gas flow at 50mL/min and subjected to a heating ramp of -20 to 110°C with a heating ramp at 10°C/min. About the TGA analysis, approximately 30mg of sample were cut and introduced into a PerkinElmer brand TGA 4000 (Akron. Ohio, USA) in an inert nitrogen atmosphere at 10ml / min, in an interval of 50 to 900°C at a speed of 10°C/min.

-Morphological Characterization

The morphological characterization of the materials was carried out by Scanning Electron Microscopy (SEM). The EDS complemented the analysis of chemical composition. Morphological analyses were performed on a FEI QUANTA 250 FEG Scanning Electron Microscope (ESEM FEI Co., Hillsboro, OR, USA). Before the experiments, they were placed on a pin and coated with gold for 25s, at 4 mA, under vacuum conditions.

-Heat transfer test In Situ

**Figure 1:**
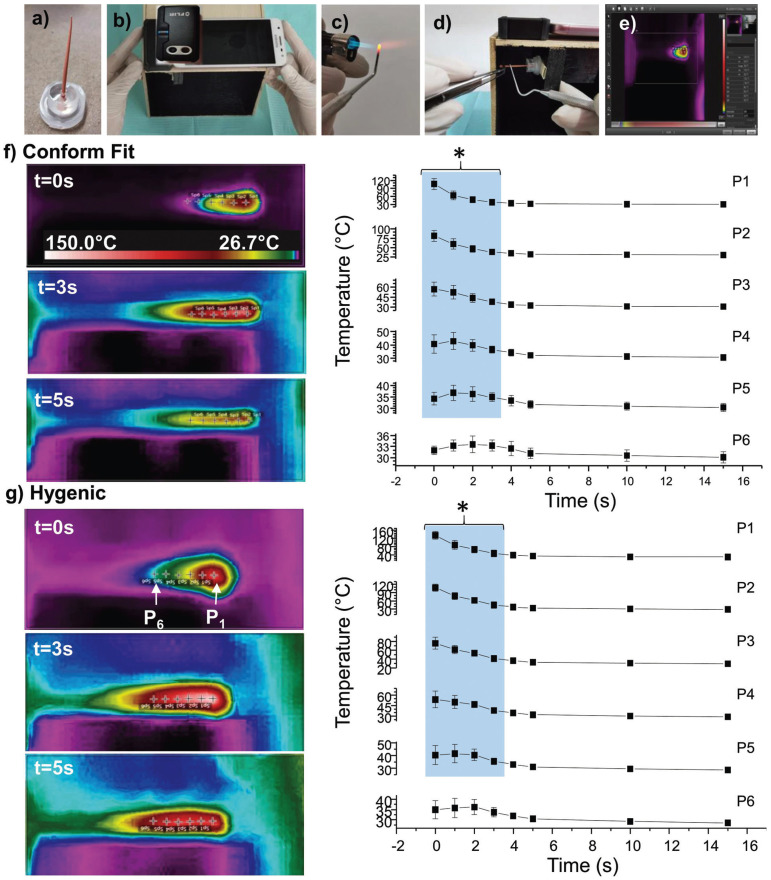
a-e) Experimental procedure for the Heat Transfer Test In Situ. a) Base of the gutta-percha cone designed in acrylic, b) System designed for the adaptation of the thermal camera (FLIR ONE® PRO by MicroUSB P/N 435-0011-01), c-d) Condenser (Hu-friedy RCP10A of 29mm) and Micro Torch used for the cutting process, e) Images obtained with the FLIR Tools program. f-g) Average temperatures in GP cones, as a function of time after cutting for each reference point (Temperature profiles).

Infrared thermography was used for in situ analysis during the cutting process of the Gp cones (n=11/group). For this, a system (black camera) was built that allowed minimizing the possibility of movement, standardization of the reference point at the time of image capture, and reduction of the influence of ambient light that could be reflected in the measurement system (Fig. 1a-e), to which an acrylic base was adhered that supports the Gp cone at the moment of cutting (Fig. 1a). The images of samples were taken with the thermal camera (FLIR ONE® PRO by MicroUSB *P* / N 435-0011-01) at 0, 1, 2, 3, 4, 5, 10, 15, and 20s after the cutting process. It is worth mentioning that the condenser instrument (Hu-friedy RCP10A of 29mm) was heated for 15s in the fire using Micro Torch Burner ST2200 Dental Packs Mex., and the climatic conditions will be established at room temperature, approximately 26±5ºC in a closed room to avoid current flow.

-Statistical analysis

Statistical analyzes were performed with SigmaPlot Version 11.0 (SPSS, Chicago, IL). For points 1 to 6 of the heat transfer profile at 0 to 5s. The Shapiro-Wilk test was used to determine the distribution of normal data. The significance of the difference in means between the two groups studied for each of the points was evaluated using Student’s t or Mann–Whitney U tests; the alpha level was set at 0.05.

## Results

**Figure 2:**
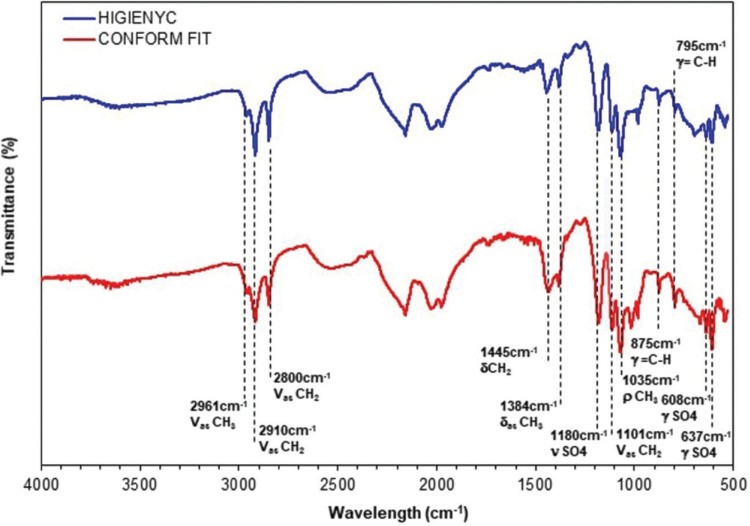
Infrared spectra of Gp Conform Fit and Hygenic.

In Figure 2, the infrared spectra obtained from the evaluated samples of the Gp Hygenic and Conform Fit are shown. The samples were measured in transmittance (%) in the mid-infrared range. In them, we can appreciate the vibrations corresponding to 1-4 cis isoprene. In other words, at 2961 cm-1, there is an intense asymmetric vibration, and at 1384cm-1, a signal of the symmetric type of deformation, both corresponding to the methyl group (CH3). Likewise, at 1035cm-1, a rocking vibration corresponding to the same group is observed (2,11,12).

On the other hand, at 2910, 2800, 1101, and 1445cm-1, signals are associated with the methylene group (CH2). While at 875 and 795cm-1, weaker signals corresponding to bending out of the double bond plane are reflected (=C-H) (2,11,12). On the other hand, vibrations related to the sulfate group are observed at 608, 637, and 1180cm-1 (13,14).

**Figure 3:**
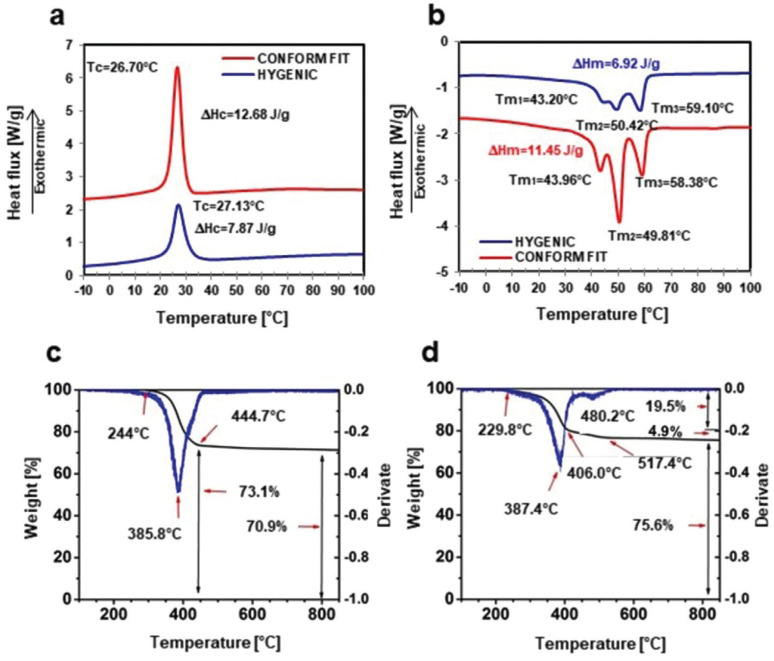
Thermal analysis. a) DSC Heating Traces, b) DSC Cooling Traces, c) Gp Conform Fit TGA, and d) Gp Hygenic TGA.

About the thermal tests, the results are presented in Figure 3. For the thermograms associated with the cooling process (Fig. 3a), an exothermic peak associated with the crystallization process of the material can be observed for both samples. However, a more pronounced and narrower peak can be seen in the Conform Fit vs. the Hygenic, which is reflected in the enthalpy associated with the crystallization process (ΔHc), as well as a slight shift in the maximum temperature of crystallization (Tc). Later in Figure 3b, the heating traces of the second cycle are presented, where three endothermic peaks associated with the fusion process can be seen, in which said fusion process of both materials is completed at approximately 60°C. As can be seen in these thermograms, there are no considerable variations in the transition temperatures, but there is a significant variation in the enthalpies of crystallization and fusion. Since the enthalpies of the Gp Conform Fit are approximately 60% higher than the enthalpies of the Gp Hygenic.

The melting process is a first-order transition, which means that when the melting point is reached, the temperature of the polymer does not increase until the phase transition from solid to liquid state is complete. That is, until the crystals present in the material melt, the enthalpy of fusion being the amount of energy necessary to complete said phase transition. Therefore, by having a higher enthalpy of fusion in Conform Fit gutta-percha cones, the transition requires more energy to be absorbed before generating a change in temperature in the material. While during the crystallization process, the material will have to release more energy before it solidifies. What has favorable clinical repercussions in working time during the obturation process. Therefore, the analysis of the thermal properties of the gutta-percha cones used as fillings in the root canal plays an important role during the obturation process. Its understanding could help the specialist to determine the appropriate obturation technique to improve the packing of the material and to eliminate all the entrance doors between the root canal.

About the thermogravimetric analysis (TGA), the results obtained from the test are presented in Figure 3c,d. We can see that the weight remains almost constant and stable up to approximately 200°C, and subsequently presents a loss in weight that concludes around 450°C. This point was used to determine the percentage by weight of fillers, and the content of polymeric material in gutta-percha (Gp) cones. About this, the Conform Fit has around 29.1% Gp and 70.9% loads (Fig. 3c). While in the case of the sample corresponding to the Hygenic, it presents around 24.4% of Gp and 75.6% in loads (Fig. 3d).

**Figure 4:**
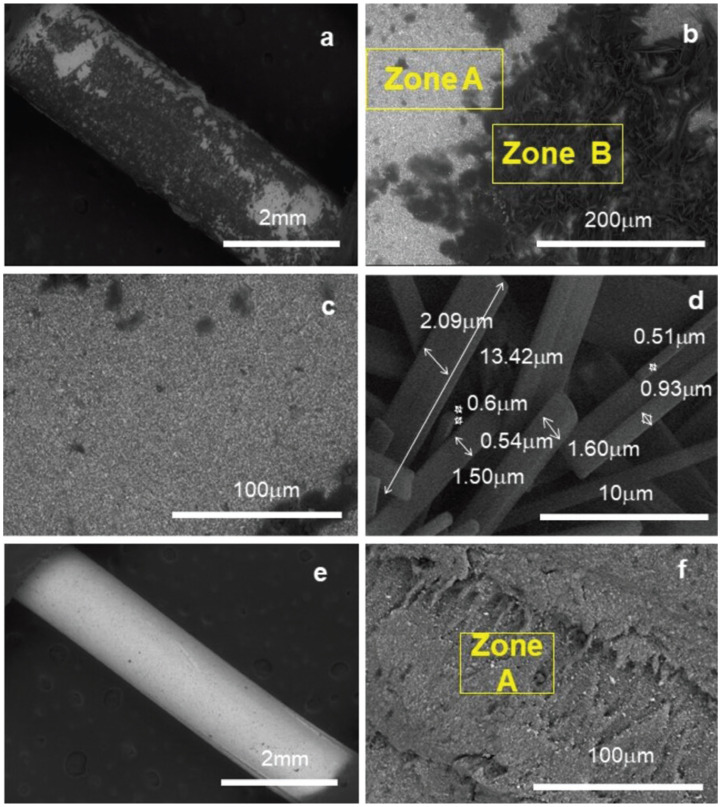
Micrographs of the Cone of Gutta-percha overview. a) Conform Fit 50x, b) Conform Fit 500x, c) Conform Fit 1000x Zone A, d) Conform Fit 10000x Zone B, e) Hygenic 50x and f) Hygenic 1000x.

The micrographs at different magnifications were evaluated for each of the Gp using backscattered electrons (BSE-SEM). In the case of Gp Conform Fit (Fig. 4 a-d), it can be seen that the material consists of a homogeneous matrix with a light microtextured coating (zone B) in the form of bars with dimensions ranging from 0.5 to 2 µm wide by 14µm long (Fig. 4d), which according to the results of the EDS analysis of each of the zones it can be said that the coating is constituted by a higher amount of organic material than concerning to the body of the Gp cone (Table 1). In relation to the microtextured coating of the Gp Conform Fit cone, it could have important clinical implications, since having a lower filler content, it would have greater fluidity, generating a better seal in the peripheral canals during the obturation process. About the Gp Hygenic cones, we can see that it is a cone constituted in a single completely homogeneous phase where elements of such as Zn, C, O, Ba, S, and Si were mainly detected.

**Table 1:**
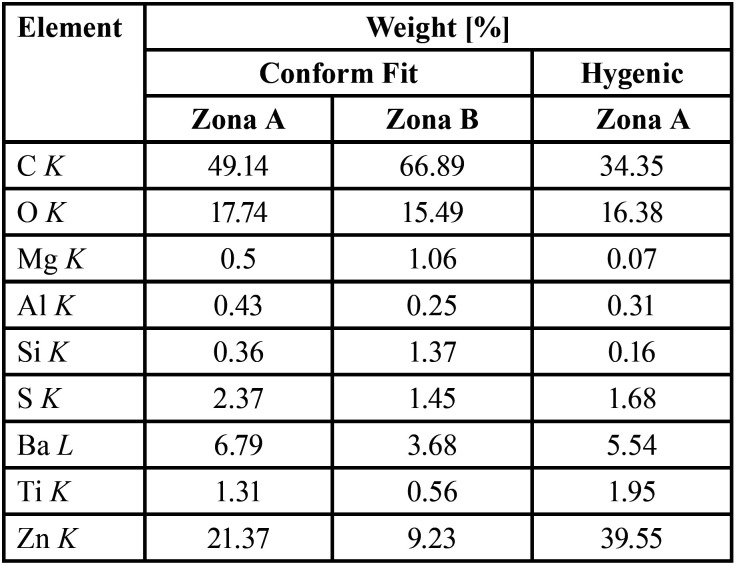
% content by weight of elements identified by SEM/EDS.

Figure 1 summarizes the results of the in situ tests, where the heat transfer at the time of cutting was evaluated. The images obtained for a cone can be seen in the left panel (Fig. 1). It shows how the temperature diffusion dissipates along the Gp cone. The images allowed the construction of temperature profiles for different selected points in different time intervals. According to the statistical analysis, with 95% confidence, a significant difference in heat transfer is determined during the first 3 s after cutting along the gutta-percha cone within points 1 to 5.

## Discussion

Research in Gp cones as an obturation material has been conducted for a long time, trying to improve their properties by modifying their composition and physicochemical properties, which guarantee a hermetic seal and efficient heat transport during the final stage of endodontics. Schilder and Buchanan (15) used the thermoplastic benefits of Gp in their obturation techniques for the root canal system, recommending plasticizing a Gp cone in the root canal and then compacting it vertically to be later filled with thermoplasticized Gp.

The article described by Ratri (12) characterized the Gp cones through means of Fourier Transform Infrared Spectroscopy (FTIR), Wide Angle X-ray Scattering (WAXS), and (Small Angle X-ray Scattering (SAXS). They evaluated the process of structural evolution of synthetic Gp polymers seen from hierarchical levels, showing the regularization of the conformation of the molecular chain and demonstrating that the α form appears sTable at high temperatures. In contrast, the α form is unsTable at high temperatures, melting at a relatively lower temperature of about 50°C. The molten sample transforms back into a crystalline form at a higher temperature. About the thermal characterization obtained in the present investigation, they agree that both materials present polymorphism. However, the results of the thermograms during the fusion process show the presence of three crystalline phases for both samples; the first around 43°C, followed by one at 50°C, and finally one at 59°C. These results coincide with those reported by Marroquín (16) in 2015, where he describes the physical properties of different types of Gp, softening approximately between 40-49°C and being flexible at approximately 61-78°C. However, he mentions that the malleability of the Gp cone occurs from temperatures above 65°C, where the material has exceeded its melting temperature. However, it should be emphasized that the temperature should not exceed approximately 230°C, since the material begins to degrade, as can be seen in the TGA analyzes.

On the other hand, with the enthalpy of fusion found in the Differential Scanning Calorimetry (DSC) analyzes, the Gp Conform Fit was determined at 11.45J/g. At the same time, for the Gp Hygenic cones, it was 6.92J/g. These results have a direct impact on heat transfer, since Gp Hygenic, having a lower enthalpy of fusion, requires less energy for the phase change (solid state to a molten state), so when applying a constant energy heat, excess energy is reflected in an increase in the final temperature at the cut-off point compared to the cones of Gp Conform Fit, which present a higher enthalpy of fusion, and thus require more energy for your phase change. Furthermore, an increase in temperature at the cut-off point is not so marked, as seen in thermal analysis with Infrared. On the other hand, in the endotherms of the cooling process, the enthalpy of fusion (∆Hc) of the Conform Fit was determined at 12.68J/g, while in the cones of the Gp Hygenic, it was 7.87J/g. Therefore, when starting from a molten state, the Conform Fit Gp cones need to release more energy to change to a crystalline state, giving us a longer working time with the Gp at the moment of obturation.

Regarding the chemical characterization, our results coincide with the infrared spectra reported by Silva and Paula in 2005 (2), where they identified the vibrations of the characteristic bands of the trans-1,4-polyisoprene polymer corresponding to the organic part of the material shutter. These spectroscopic characteristics is present in both brands of Gp evaluated. However, the complementary analyzes of TGA and EDS helped in the chemical characterization of the materials. In which differences of up to 5% of its composition were found for the content of Gp polymeric material. Likewise, solids can be associated with the presence of barium sulfate (BaSO4), as well as the content of metal oxides that provide rigidity, consistency, radiopacity, preservation, antisepsis, and coloration to the material.

 In such a way that the Gp Conform fit cone has approximately 30% by weight of poly (trans-1,4 isoprene) thermoplastic material, and the Gp Hygenic cone 25% by weight. These results could influence the three-dimensional sealing of the material due to plasticity; in such a way, *in vitro* studies should be evaluated. However, these variations in compositions resemble those found by Ferreira *et al*. (5)

## Conclusions

The characterization of the materials was useful to observe variations in the composition and configuration of two commercial brands of Gp cones. These variations directly influence the response of the material to thermal phenomena in the first 3 seconds after the cutting process due to the chemical composition of the material which affects heat transfer phenomena.

## References

[1] Vishwanath V, Rao HM (2019). Gutta-percha in endodontics-A comprehensive review of material science. J Conserv Dent.

[2] Silva-Júnior JBA, Paula RD, Feitosa JPA, Gurgel-Filho ED, Maniglia-Ferreira C, FJ Filho DS (2006). In vivo aging of gutta-percha dental cone. J Appl Polym Sci.

[3] Dong M, Zhang J, Liu L, Hou G, Yu Y, Yuan C (2019). New gutta percha composite with high thermal conductivity and low shear viscosity contributed by the bridging fillers containing ZnO and CNTs. Compos B Eng.

[4] Liao SC, Wang HH, Hsu YH, Huang HM, Gutmann JL, Hsieh SC (2021). The investigation of thermal behaviour and physical properties of several types of contemporary gutta-percha points. Int Endod J.

[5] Maniglia-Ferreira C, Gurgel-Filho ED, Silva Jr JBA, Paula RCMD, Feitosa JPA, Gomes BPFDA (2007). Brazilian gutta-percha points. Part II: thermal properties. Braz Oral Res.

[6] Roberts HW, Kirkpatrick TC, Bergeron BE (2017). Thermal analysis and stability of commercially available endodontic obturation materials. Clin Oral Investig.

[7] Cavenago BC, Ordinola-Zapata R, Duarte MAH, del Carpio-Perochena AE, Villas-Bôas MH, Marciano MA (2014). Efficacy of xylene and passive ultrasonic irrigation on remaining root filling material during retreatment of anatomically complex teeth. Int Endod J.

[8] Hsu YH, Wang HH, Shen YK, Gutmann JL, Hsieh SC (2020). Thermal behavior and viscoelastic properties of gutta-percha used for back-filling the root canal. J Dent Sci.

[9] Lipski M, Woźniak K (2003). In vitro infrared thermographic assessment of root surface temperature rises during thermafil retreatment using system B. J Endod.

[10] Atmeh AR, Hadis M, Camilleri J (2020). Real-time chemical analysis of root filling materials with heating: guidelines for safe temperature levels. Int Endod J.

[11] Gavish M, Corrigan J, Woodward AE (1988). FTIR investigations of crystallinity and surface reaction for trans-1, 4-polyisoprene lamellar structures crystallized from solution. Macromolecules.

[12] Jaya-Ratri P, Tashiro K (2013). Application of the simultaneous measurement system of WAXD, SAXS and transmission FTIR spectra to the study of structural changes in the cold-and melt-crystallization processes of trans-1, 4-polyisoprene. Polym J.

[13] Sun Y, Zhang F, Wu D, Zhu H (2014). Roles of polyacrylate dispersant in the synthesis of well-dispersed BaSO4 nanoparticles by simple precipitation. Particuology.

[14] Ramaswamy V, Vimalathithan RM, Ponnusamy V (2010). Synthesis and characterization of BaSO4 nano particles using micro emulsion technique. Adv Appl Sci Res.

[15] Buchanan LS (1994). The continuous wave of condensation technique: a convergence of conceptual and procedural advances in obturation. Dent Today.

[16] Marroquín BB, Wolf TG, Schürger D, Willershausen B (2015). Thermoplastic properties of endodontic gutta-percha: a thermographic in vitro study. J Endod.

